# Hallucinations as a trauma-based memory: implications for psychological interventions

**DOI:** 10.3389/fpsyg.2015.01262

**Published:** 2015-09-15

**Authors:** Craig Steel

**Affiliations:** School of Psychology and Clinical Language Sciences, University of Reading, Reading, UK

**Keywords:** hallucinations, posttraumatic stress disorder, intrusive memory, voices, visions

## Abstract

The relationship between hallucinations and life events is a topic of significant clinical importance. This review discusses the extent to which auditory and visual hallucinations may be *directly* related to traumatic events. Evidence suggests that intrusive images occur frequently within individuals who also report hallucinatory experiences. However, there has been limited research specifically investigating the extent to which hallucinations are the re-experiencing of a traumatic event. Our current theoretical understanding of these relationships, along with methodological difficulties associated with research in this area, are considered. Recent clinical studies, which adopt interventions aimed at the symptoms of posttraumatic stress disorder in people diagnosed with a psychotic disorder, are reviewed. There is a need for the development of evidence-based interventions in this area.

## Introduction

The idea that stressful and traumatic life events may be relevant to the content of hallucinatory experiences is not a new one. Freud (1936) argued that the phenomenon of hallucinations was a product of forgotten or repressed traumatic memories entering the conscious mind. Naturally, as the disciplines of psychology and psychiatry develop, our understanding of these relationships also develops. This review discusses the extent to which the content of auditory and visual hallucinations, hereafter referred to as voices and visions, may be *directly* related to traumatic events. Implications for current clinical practice and future research are also discussed.

Recently, there has been increased awareness of the prevalence of traumatic life events within individuals diagnosed with schizophrenia (see Grubaugh et al., 2011, for a review). It has been estimated that 15% of this group will also be suffering from posttraumatic stress disorder (PTSD; Achim et al., 2011). However, there is debate as to whether these diagnoses are distinct conditions or whether the same phenomena may fulfill the diagnostic criteria for both disorders. For example, hearing voices/seeing visions may be both a hallucination, in terms of the diagnostic criteria for schizophrenia, and categorized as the re-experiencing of a traumatic event with respect to PTSD. At this point it is worth noting that the hallmark symptom of PTSD is considered an intrusive memory of a traumatic event, most likely in the form of a visual image. Whereas, with respect to schizophrenia, the intrusive sensory phenomena that have received most attention are in the auditory modality, i.e., hearing voices. Consequently, most of the research aimed at understanding psychotic phenomena with respect to traumatic events has focussed on hearing voices. Whereas theoretical developments drawing on psychological models of PTSD have predominantly focussed on intrusive images.

## Linking the Content of Hallucinations with the Content of Trauma Memories

An early study in this field involved the assessment of case records of 100 patients admitted to a psychiatric unit. Read and Argyle (1999) report that approximately half the symptoms for which content was recorded appeared to be related to childhood abuse. However, with respect to hearing voices, the available data was limited to seven patients. Morrison et al. (2002) interviewed 35 people with a diagnosis of schizophrenia and reported that 74.3% were able to identify an image in relation to their psychotic symptoms and, of those, 70.8% (*n* = 17) made an explicit link between the image and a particular event in their past. Examples include an individual who had been raped and sexually abused as a child, reporting an intrusive image of a bearded man shouting. In this study individuals were required to report if their images were “associated” with a past event, so it is unclear whether the content was a direct re-experiencing.

The largest phenomenological survey of auditory hallucinations to date involved interviewing 199 voice hearers (McCarthy-Jones et al., 2014). Of these, 12% reported that they heard voices which were identical replays of memories of previous conversations that they had heard, whilst 31% reported that the relationship was similar but not identical. However, the previous conversations being assessed were not necessarily stressful or traumatic experiences. Reiff et al. (2011) assessed 30 individuals for potential relationships between childhood abuse and adult psychotic symptoms. They reported that individuals who had suffered abuse scored high on their measure of “trauma related content score.” However, this measure contained nine items only one of which assessed a direct memory of a traumatic event. The other items referred to a wider range of emotional and content links with hallucinations and delusions. These “thematic” links can be clinically observed at the level of emotional experience, for example having suffered a humiliating traumatic event and also experiencing humiliation in relation to current voice hearing experiences. Raune et al. (2006) also focussed on thematic associations and highlighted a specific relationship between persecutory voices and intrusive traumatic events.

One study specifically aimed to identify the prevalence of both direct and indirect relationships between traumatic events and the content of voices. Hardy et al. (2005) extracted a short amount of information describing the main traumatic event and hallucinatory content, which were then rated as either having a direct link, an indirect (emotional) link or no link at all. Based on 40 participants, 12.5% of hallucinations contained content which was a direct replication of trauma content, with 45% rated as having an emotional link.

The paucity of studies in this area may, in part, be due to the methodological difficulties involved when assessing a direct link between the content of a current intrusive sensation (voice or vision) and the content of a past traumatic event. Regardless of diagnostic disorder, traumatic memories are often formed of information which is not an identical representation of the experienced stressful event (Conway and Pleydell-Pearce, 2000). This issue is likely to exacerbate the potential for confusion when an individual experiences fleeting highly emotive intrusions and attempts to locate the origin of these phenomena. The need to rely on self-report data in these studies is therefore a major limitation.

## Theoretical Accounts of Trauma Memories and Hallucinations

In order to understand the process through which intrusive trauma-related memories may form the basis of the voices and visions associated with a psychotic episode, clinical researchers have drawn on psychological models of posttraumatic stress disorder. Steel et al. (2005) focus on information-processing models of PTSD which refer to a shift in information processing style during stressful and traumatic events (Ehlers and Clark, 2000; Brewin, 2001). Routine information processing involves a process called “contextual integration” whereby detailed encoding of incoming stimuli takes place, which facilitates later memory recall. For example, being able to remember who was at your birthday by recalling a full contextual image of the event. Also, being able to remember what happened before, and after, a specific event due to the integration of relevant temporal information. However, during a traumatic event, the need for a quick behavioral response requires rapid information processing. This is achieved through a temporary decrease in contextual integration and a quick response based on a basic perceptual processing. The provision of a rapid response to danger has the consequence of a vulnerability to experiencing intrusive memories of the stressful event at a future date. The reduced contextual processing of the trauma stimuli makes them difficult to recall on a voluntary basis, due to the lack of integration with cues which normally facilitate recall. However, the information is typically triggered involuntarily by stimuli which have a perceptual match to some aspect of the traumatic event (e.g., the smell of an attacker’s aftershave on someone else). Therefore, within these theories, reduced levels of contextual integration are considered central to the development of trauma-related intrusive memories. Thereafter, the intrusions may be maintained through a number of cognitive and behavioral processes, such as avoidance, in order to develop a presentation of PTSD. However, in order to be diagnosed with PTSD the individual, or their clinician, is likely to be confident that the intrusions are in fact memories of a stressful event.

Steel et al. (2005) note that individuals who score high on schizotypal personality questionnaires exhibit reduced levels of contextual integration when they are not stressed. It is argued that the consequent intrusions contribute to the unusual beliefs and experiences which are characteristic of high schizotypal personality. Exposure to traumatic events is likely to result in more frequent and distressing intrusive trauma-related memories. Crucially, it may be particularly confusing for these individuals to identify these intrusive phenomena as a memory. Given schizotypal personality is associated with unusual beliefs such as clairvoyance and telepathy, they may be predisposed to making sense of highly emotive intrusive phenomena within the context of these beliefs. For example, a short extract of speech may be given an external origin, thus becoming a voice hearing experience. Or a visual intrusive memory of being attacked may be interpreted as a vision, or premonition, of a planned future attack. The latter example highlights how the interpretation of intrusive memories may contribute to the development and maintenance of a number of psychotic symptoms, including paranoia (see Morrison, 2001). This is supported by a recent report showing that 73% of people classified as suffering from persecutory delusions experienced recurrent intrusive threatening images (Schulze et al., 2013). However, this study did not explicitly assess links between intrusive images and stressful life events.

Evidence supporting this account has been derived from laboratory experiments and clinical settings. Individuals who score high on schizotypal personality questionnaires report experiencing more frequent intrusions after having watched a stressful film in the laboratory (Holmes and Steel, 2004). High schizotypes also report more frequent distressing intrusions after being involved in a road traffic accident (Steel et al., 2008), and when awaiting treatment within a specialist psychology trauma service (Marzillier and Steel, 2007). It should be noted that in these studies the participants reported intrusions that contained a content match with the stressful or traumatic event they had experienced, and thus they recognized the intrusion as a memory. Further, poor contextual memory has been associated with more vivid and detailed intrusive images within psychosis-prone individuals (Glazer et al., 2013). Jones and Steel (2012), using a modified version of a free association task, reported that schizotypal personality was associated with an increased vulnerability to experience involuntary autobiographical memories.

With reference to the process in which individuals may become confused as to the origin of intrusive memories, Larøi et al. (2005) provide evidence of those scoring high on hallucination proneness (a subgroup of positive schizotypal personality) being particularly likely to make source monitoring errors. However, this relationship is still to be tested in relation to stressful or traumatic memories.

Waters et al. (2006) also refer to the role of contextual memory in the formation of hearing voices. They argue that deficits in intentional inhibition and contextual memory result in the unintentional activation of memories, which without contextual cues, are not recognized as such. The authors present experimental evidence for the role of intentional inhibition and suggest a number of potential explanations as to the relationship with hallucinatory experiences. An important difference, therefore, between this account and that of Steel et al. (2005) is that the former is not based specifically on traumatic events forming the memories which may be experienced as a voice.

The theoretical accounts discussed above would indicate a specific link between intrusive memories and the positive symptoms of psychosis. Of interest, there have been a number of recent reviews reporting on this topic. These studies provide conflicting conclusions with Bentall et al. (2014) suggesting a specific link between childhood sexual abuse and hallucinations, whilst van Dam et al. (2015) argue that childhood trauma is not related to a differential course of symptoms. However, with reference to the role of intrusive memories, these phenomena may contribute not just to voices, but also to visions and paranoid experiences. Therefore, the lack of specificity reported by van Dam et al. (2015) does not negate the role of intrusive memories within a range of symptoms.

## The Role of Dissociation

Psychological models of posttraumatic stress disorder also refer to the role of peritraumatic dissociation in the development of traumatic memories. It is therefore of interest that the tendency to dissociate has been associated with an increased likelihood to hear voices within a number of studies (e.g., Schafer et al., 2008; Perona-Garcelan et al., 2012a). Although our theoretical understanding of dissociation is limited, there is likely to be some overlap in the processes of reduced contextual integration and increased levels of perceptual processing, with the presence of dissociative states. With reference to Steel et al. (2005), this overlap is at the level of schizotypal personality and dissociation. That is, dissociative experiences can be considered a form of unusual experience and, as such, they form part of the beliefs and experiences which comprise schizotypal personality. Accordingly, Laposa and Alden (2008) report a high level of trait dissociation to be a vulnerability factor for more frequent intrusions within the previously discussed stressful-film paradigm employed by Holmes and Steel (2004). A recent study by Varese et al. (2012) indicates that dissociation positively mediates the effect of childhood trauma on hallucination-proneness within individuals diagnosed with schizophrenia spectrum disorders. With reference to a population of military veterans diagnosed with PTSD, Brewin and Patel (2010) found that those who reported hearing voices also reported higher levels of dissociation. It should be noted that these studies used the Dissociative Experiences Scale (DES; Bernstein and Putnam, 1986), which is a measure of trait dissociation and not a measure of dissociation at the time of the traumatic event. Longitudinal studies are therefore required. Escher et al. (2002) followed up a sample of 80 adolescents and found the trait dissociation (as measured by the DES) was a significant predictor of voice persistence over a 3-year period.

Few studies have attempted to disentangle the roles of dissociation and schizotypy in the development of psychotic symptoms. The previously reported study by Steel et al. (2008) suggests that schizotypal personality is the primary driver in the development of trauma-related intrusions, which may form the basis of some psychotic experiences. However, Altman et al. (1997) argue that “subclinical auditory hallucinations” have an especially strong link with dissociation, whereas “subclinical delusions” were related to schizotypal cognitions.

Our current understanding of the role of dissociation in the development of hearing voices and seeing visions is restricted due to the need for theoretical development in our understanding of dissociation itself. Many researchers argue that dissociation is not a unitary construct, and there has been some theoretical speculation as to whether the phenomenon can be split into two forms, namely detachment and compartmentalization (Holmes et al., 2005; Brown, 2006). Adopting this approach, Perona-Garcelan et al. (2012b) found that the depersonalization component of dissociation, a form of detachment, was the mediator between childhood trauma and hearing voices. They also identified that this relationship was specific to hallucinations and not delusions.

In summary, existing research suggests that individuals scoring high in trait schizotypy are also likely to score high on trait dissociation. Accordingly, these individuals are prone to higher levels of state dissociation during stressful and traumatic events. Dissociation, at an information processing level, is associated with a breakdown in contextual processing. This style of information processing leaves an individual vulnerable to an increased level of trauma-related intrusive memories (Steel et al., 2005; Waters et al., 2006), which may be appraised in a manner consistent with the experience of hearing voices or seeing visions.

## Clinical Implications

Although the prevalence of PTSD is estimated at 15% in people diagnosed with schizophrenia, there is only one published randomized controlled trial which targets this “co-morbid” group. van den Berg et al. (2015) report that both Eye movement desensitization and reprocessing (EMDR) and exposure interventions were successful in reducing the severity of posttraumatic stress symptoms in this population. However, reduced levels of PTSD symptoms were not associated with a reduction in hallucinatory experiences (van der Gaag, 2015) suggesting a less direct link between these phenomena than suggested within the theoretical accounts discussed above.

There are no reports on the explicit targeting of dissociation within individuals who hear voices or see visions. As discussed, our limited theoretical understanding of dissociation may have restrained clinical development in this field. However, the phenomena has been discussed in the context of individuals being formed of multiple selves, rather than a single personality construct. It has been argued that the content of voices may originate from a breakdown in the integration of these multiple selves, with more than one self being heard at one time (Longden et al., 2012). This perspective forms the basis of the Voice Dialogue approach, within which a voice hearer is encouraged to open a verbal dialogue with their voices (other selves) in order to work toward a more functional relationship with them (Corstens et al., 2011).

Neither of the interventions discussed so far are based on targeting a direct link between traumatic events and the content of voices and visions. Current reports of the prevalence of such links are likely to be an underestimate given the methodological difficulties discussed earlier, and the tendency for links not to be noticed rather than identified in error. This issue is also relevant during clinical assessment. Morrison (2001) highlights how the appraisal of an intrusive experience may impact on whether an individual is diagnosed with an anxiety disorder or a psychotic disorder. The latter being characterized by “socially-unacceptable” interpretations of their intrusive experiences, e.g., the government are putting these pictures in my head using a machine. Further, Steel et al. (2005) describe how existing unusual, or schizotypal, beliefs are likely to contribute to this type of appraisal. The role of appraisal is a key process in cognitive behavior therapy, and as such has been adopted within a large number of clinical trials. A small to moderate effect size has been reported for this generic intervention in relation to hearing voices (van der Gaag et al., 2014). There would, therefore, seem to be room for improvement. It is possible that interventions which target intrusive memories, where the content of psychotic symptoms directly relate to a traumatic event, may, where relevant, be more effective.

To date there has been little clinical development in this area. The most relevant interventions would seem to be those developed for use with distressing intrusive experiences associated with PTSD, such as “reliving,” or exposure to the original traumatic event. Recent studies suggest acceptability and effectiveness when using exposure with people diagnosed with schizophrenia (Frueh et al., 2009; van den Berg et al., 2015). It is also worth noting that even high levels of dissociation do not have an adverse impact on the outcome of this intervention within a non-psychotic population (Hagenaars et al., 2010). Another intervention originating from the field of trauma is “imagery rescripting.” The aim is to modify the meaning of the intrusive memory by first imagining the accurate memory and then rewriting a new ending to the narrative. Rescripts typically include the individual gaining more control, or arriving at a peaceful resolution to an unresolved emotional situation. Whilst first developed for use with survivors of childhood sexual abuse (Smucker et al., 1995), it has recently been adopted for use with a range of different disorders. Unlike reliving this intervention does not require prolonged exposure to an emotionally distressing memory. This is likely to contribute to lower drop-outs during an imagery rescripting intervention than an exposure intervention (Arntz et al., 2013).

At the time of writing, there is one published study reporting the use of imagery rescripting with individuals who hear voices (Ison et al., 2014). The authors report a small case series in which they successfully focused on rescripting repetitive intrusive memories, however the content of the memory was not necessarily explicitly linked to the content of voices. Given that a recent study indicates that imagery rescripting is a highly acceptable and effective intervention when working with reactions to complex trauma (e.g., Arntz et al., 2013) future research applied to distressing voices and visions would seem a priority.

In summary, the relationship between hearing voices, seeing visions and traumatic events continues to intrigue clinical researchers. However, a full theoretical understanding remains elusive. The subgroup of voices and visions which are directly linked to stressful events are likely to be responsive to interventions which are based on a well developed theoretical understanding of reactions to trauma.

### Conflict of Interest Statement

The author declares that the research was conducted in the absence of any commercial or financial relationships that could be construed as a potential conflict of interest.
